# Heterozygous *Men1*(+/T) Knockout Mice Do Not Develop Bronchopulmonary Neuroendocrine Hyperplasia or Neoplasia but Bronchial Adenocarcinoma

**DOI:** 10.3390/arm93020007

**Published:** 2025-03-31

**Authors:** Max B. Albers, Ludger Fink, Jerena Manoharan, Caroline L. Lopez, Carmen Bollmann, Detlef K. Bartsch

**Affiliations:** 1 Department of Visceral, Thoracic and Vascular Surgery, University Hospital of Giessen and Marburg, Philipps University of Marburg, 35043 Marburg, Germanybartsch@med.uni-marburg.de (D.K.B.); 2 Institute of Pathology, Dermatopathology, Cytology and Molecular Pathology, UEGP, 35578 Wetzlar, Germany; fink@patho-uegp.de

**Keywords:** MEN1, multiple endocrine neoplasia, bronchial neuroendocrine neoplasia, bronchial adenocarcinoma

## Abstract

**Highlights:**

Patients with the multiple endocrine neoplasia type 1 (MEN1) syndrome are at risk of developing tumors of the endocrine pancreas, hyperparathyroidism, and pituitary adenomas, among tumors of other organs and non-tumorous manifestations. In humans, bronchopulmonary neuroendocrine neoplasias are a rare manifestation of the MEN1 syndrome with a penetrance of 2–7%. Due to the rarity of the syndrome, this study aimed to describe pulmonary changes and identify precursor lesions of bronchopulmonary neuroendocrine neoplasias and the potential prophylactic effect of somatostatin analogues in a well-established heterozygous *Men1* knockout mouse model. None of the mice in this study, somatostatin analogue-treated, or placebo-treated, developed neuroendocrine neoplasias nor precursor lesions. However, 10% of the individuals developed bronchial adenocarcinoma, which has not been described to be part of the human MEN1 syndrome to date and needs to be evaluated in future investigations.

**What are the main findings?**

- Heterozygous *Men1*(+/T) knockout mice do not develop bronchopulmonary neuroendocrine hyperplasia or neoplasia.
- Ten percent of *Men1*(+/T) knockout mice develop bronchial adenocarcinoma, a condition not previously linked to human MEN1-syndrome.

**What is the implication of the main finding?**

- The first main finding elucidates a weakness of this animal model, which has been described as well comparable to the human MEN1 syndrome in earlier studies.
- The occurrence of bronchial adenocarcinoma in these mice warrants further investigation into its potential association with human MEN1 syndrome.

**Abstract:**

Introduction: Bronchopulmonary Neuroendocrine Neoplasms (NEN) occur in 2–7% of patients with multiple endocrine neoplasia type 1 (MEN1). Precursor lesions have been identified for MEN1-related pancreatic, duodenal, and gastric NEN. The aim of the current study using a MEN1 mouse model was to define the precursor lesions of bronchopulmonary NEN and evaluate the potential prophylactic antitumor effects of somatostatin analogues in a transgenic MEN1 mouse model. Methods: Fifteen mice, germline heterozygous for *Men1*(+/T), were treated with subcutaneous injections of lanreotide autogel (Somatuline Autogel^®^, IPSEN Pharma), while 15 mice were treated with subcutaneous injections of physiologic sodium chloride as the control group. Five mice from each group were euthanized after 12, 15, and 18 months, respectively. The complete lungs were resected and evaluated after hematoxylin and eosin staining and immunohistochemistry for synaptophysin and chromogranin A. Results: In the lungs of the 30 evaluated mice, whether treated or placebo treated, no bronchopulmonary neuroendocrine cell hyperplasia nor neuroendocrine neoplasia was detected through histopathology. However, pulmonary adenocarcinoma developed in 2 (13%) of the 15 untreated mice and in 1 (7%) of the 15 lanreotide-treated mice. Conclusions: Heterozygous *Men1*(+/T) knockout mice do not develop bronchopulmonary NEN or precursor lesions, but pulmonary adenocarcinoma. This surprising result needs to be investigated in more detail.

## 1. Introduction

Multiple endocrine neoplasia type 1 (MEN1) is caused by a heterozygous germline mutation of the *MEN1* gene on chromosome 11q13 [1]. Patients with MEN1 are at risk of developing neuroendocrine neoplasms (NEN) in the pancreas, duodenum, and pituitary. They are also at risk of hyperparathyroidism, as well as other associated tumors, including adrenal cortical adenomas, thymic, gastric, and bronchopulmonary NEN [2,3,4]. Bronchopulmonary NEN is a less common manifestation of MEN1. Their penetrance was estimated to be approximately 2–7% in clinical observations [2,3,4]. However, small nodular tumors of more than 3 mm could be detected by high-resolution imaging in approximately 30% of MEN1 patients, suggesting that bronchopulmonary NEN might be more common than previously recognized [2]. Due to their rarity, the natural course and optimal treatment of MEN1-associated bronchopulmonary NEN remain poorly understood [5]. In accordance with pancreatic NEN, most experts indicate surgery for tumors exceeding 20 mm in diameter to prevent either metastatic spread or local complications, such as bleeding or bronchial obstruction [2,5]. Although an aggressive course of surgically treated bronchopulmonary NEN is rare, metastatic spread and lethal outcomes of aggressive tumor disease have been described, and recurrent or bilateral lung surgery may constitute a heavy burden for affected patients [2,3].

It could be shown that pancreatic, duodenal, and gastric NEN are preceded by a distinct type of neuroendocrine cell hyperplasia [6,7], which might be a target for prophylactic pharmacological treatment. In one patient series published in 2016 [2], among four re-evaluated specimens of resected MEN1-associated bronchopulmonary NEN, multifocal intrapulmonary areas of neuroendocrine cell hyperplasia were found in the surrounding lung tissue of three patients, which might represent precursor lesions for bronchial NEN. Preclinical studies [8] as well as first observational studies in patients with MEN1 [9] suggest that somatostatin analogues (SSA) might be effective for primary and secondary prevention of MEN1-associated pancreatic NEN.

The antitumor effect of SSAs is achieved through both direct and indirect mechanisms, involving the modulation of intracellular signaling pathways, inhibition of cell proliferation, apoptosis, and suppression of growth factors and angiogenesis. While somatostatin binds to all five receptor subtypes, lanreotide has a predominant affinity to somatostatin receptor (SSTR) 2, which both inhibits cell proliferation and mediates apoptosis via the MAP kinase signaling pathway [10]. Although the majority of lung adenocarcinomas express SSTR (predominantly SSTR 5) [11], the use of SSA has not been reported to have a relevant clinical benefit in lung adenocarcinoma.

Since MEN1-associated NEN behave differently from sporadic NEN and the rarity of the syndrome as well as the required long follow-up periods hampers the viability of clinical studies, a heterozygous *Men1*(+/T) mutant mouse model has been established [12]. The protein encoded by the murine *Men1* gene has a close similarity of 97% to the human tumor suppressor protein MENIN, and heterozygous *Men1*(+/T) knockout mice develop various neuroendocrine neoplasms mimicking the MEN1 syndrome in humans [12]. Several groups, including the authors, described the development of pancreatic, duodenal, parathyroid, and pituitary tumors [8,12,13,14,15]. To date, bronchopulmonary NEN have not been reported in this model.

The aim of this study was to describe pulmonary findings and evaluate the development of bronchopulmonary NEN and their precursor lesions in a preclinical model of *Men1*(+/T) heterozygous mutant mice as well as the potential preventive effect of lanreotide.

## 2. Materials and Methods

### 2.1. Mouse Model

The mouse model used for this study was a friendly gift to our laboratory from Philippe Bertolino and Chang Zhang from the Faculty of Medicine, University of Lyon, France. In these mice from a mixed genetic background (129/Sv X C57BL/6), the murine *Men1* gene was disrupted by the gene targeting technique. While homozygous *Men1*(T/T) mice are not viable, heterozygous *Men1*(+/T) mice appear normal at a young age and develop numerous tumors of neuroendocrine origin until the age of 18 months [16]. In detail, until the age of 18 months, *Men1*(+/T) mice from the original study by Bertolino et al. [12] developed hyperparathyroidism (41%), pancreatic islet hyperplasia or pancreatic NEN (88%), pituitary adenoma (6%), pituitary carcinoma (13%), adrenocortical hyperplasia or adenoma (35%), and also a high number of testicular tumors (59%) and sex-cord stromal tumors (31%). Bronchopulmonary NEN have not been reported by this group.

Many of these manifestations are typical of the human MEN1 syndrome. However, the penetrance rates of the typical manifestations as well as the high incidence of testicular and sex-cord tumors also demonstrate significant differences between human and murine effects of the heterozygous loss of the tumor suppressor protein MENIN [13].

### 2.2. Treatment

All experiments were performed in compliance with the Animal Research: Reporting of In Vivo Experiments (ARRIVE) guidelines and after approval by the local committee for animal care (Regierungspräsidium Gießen, Dezernat 54; V54-19c2015 h 01 MR 20/16 Nr. 77/2013). Mice were raised in a controlled environment at 22.6–23.0 °C at a 12-h day-night rhythm and fed ad libitum with a standard rodent diet. Genotyping was performed from the DNA of the mice’s tail biopsy at the age of 14 days using a standard PCR protocol. The used primers’ sequences were 3f1, 5′-GGATTCTGCCCCAGGC and 3r1, 5′-CACCTCCATCTTACGGTCG, as previously published by our group [14].

Overall, 30 *Men1*(+/T) mice were included in the study. Of these, 15 were treated with subcutaneous injections of lanreotide (Somatuline*^®^* autogel, IPSEN pharma, Paris, France) starting at the age of five weeks. Per injection, 100 µg of lanreotide were diluted with aqua ad injectable to a volume of 200 µL and administered subcutaneously in the nuchal fold. Injections were repeated once every 28 days until the euthanizing. The remaining 15 mice were treated as a control group with subcutaneous injections of physiologic sodium chloride. Within both the control group and the lanreotide-treated group, 8 mice were female. Five mice of each group were euthanized at the ages of 12, 15, and 18 months, respectively. For Euthanasia, cervical dislocation was performed by trained personnel after sedation with intraperitoneal injection of 5 µg/g midazolam. The resected lungs were examined for macroscopically visible tumors, fixed in formalin, and embedded in paraffin blocks. In one mouse with a macroscopically visible tumor, the tumor was carefully separated from the surrounding tissue and transected separately. For all other cases, both lungs were embedded together in a single block and fully sectioned to ensure representation of tissue from both lungs.

### 2.3. Tissue Staining

The slides were deparaffinized using xylol (twice for ten minutes) and rehydrated with 100%, 95%, 80%, and 60% ethanol for five minutes each. All tissue sections were stained with hematoxylin and eosin (H&E). Hematoxylin and Prussian blue staining were used to confirm the presence of hemosiderin deposits. Elastica van Gieson stain was applied to tumor sections.

Immunohistochemistry was performed strictly following the antibody manufacturer’s recommendations. Target protein retrieval was achieved by microwaving the slides for 10 min in 10 mmol/L sodium citrate buffer (pH6.0), followed by tissue blocking with H2O2-methanol (3%) for 15 min at room temperature. The tissues were then washed with distilled water and PBS. Incubation with the primary antibody was performed overnight at 5 °C. The antibodies used included rabbit-derived monoclonal synaptophysin antibody (Invitrogen Synaptophysin Monoclonal Antibody SP11, ThermoFisher Scientific, Waltham, MA, USA) and polyclonal Rabbit anti-CgA antibody (Invitrogen Chromogranin A Polyclonal Antibody PA5-85952, ThermoFisher Scientific, Waltham, MA, USA) at a 1:100 and 1:1000 dilution. After washing with PBS, an HRP-conjugated anti-rabbit secondary antibody was applied, and the slides were incubated for one hour at room temperature. The slides were then washed with PBS and developed using the ABC Elite Kit (Vector Labs, Burlingame, CA, USA) according to the manufacturer’s instructions. Counterstaining was performed with hematoxylin. For the reproduction of the results, the immunohistochemistry for both synaptophysin and CgA was repeated with a higher concentration of the primary antibody of 1:50 and 1:750, respectively. To rule out the possibility of faulty staining, the repeated immunostaining against synaptophysin was performed using an on-slide control with a pancreatic section positioned next to each section of the lung on the same object slide.

Microscopic examination was performed by the first author (MBA) and a specialist for lung pathology (LF).

## 3. Results

All 30 mice reached the target age of 12, 15, or 18 months and were sacrificed according to the study protocol. The development of neuroendocrine tumors typical for the mouse model was confirmed by processing the pancreata of the mice, as published in [8].

### 3.1. Macroscopic Examination

Macroscopic examination of a 15-month-old lanreotide-treated mouse revealed a 4 mm large, firm, white-greyish tumor with a smooth surface in the right lung, which infiltrated about half of the lung volume. Due to its size, infiltrative growth, and central location, the tumor could not be assigned to one of the four lobes of the lung. The tumor was dissected and embedded separately. Otherwise, there were no macroscopic abnormalities, in particular no pulmonary or mediastinal masses.

### 3.2. Histopathological Examination

**Figure 1:**
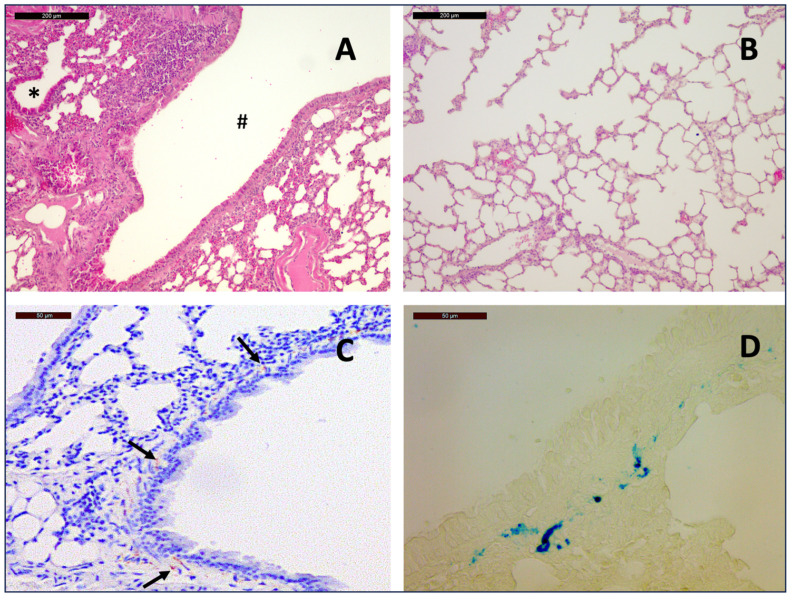
General histopathological findings: Hematoxylin and Eosin staining of a section from the lung of a 12-month-old mouse at 10× magnification shows lymphocytic infiltration around the bronchi (#) and bronchioles (*) (**A**) as well as rarefication of alveolar septa (**B**), indicative of chronic bronchitis and emphysema. Hemosiderin deposits among smooth muscle cells of the bronchial walls (arrows) as seen on slides with immunostaining for Chromogranin A (**C**) could be distinguished from immunostaining by the pattern of distribution and by Prussian blue staining ((**D**), 40×)).

In the control group and the SSA-treated group, 12 of 15 mice (80%) and 11 of 15 mice (73%), respectively, showed lymphocytic infiltrations around the bronchi and bronchioles, along with mild fibrosis of the bronchial wall, indicative of chronic bronchitis (Figure 1). Additionally, mild rarefaction of the alveolar septa was observed, which, in combination with chronic bronchitis, might be an indicator for emphysema. Apart from this, no abnormalities were found in the general histological morphology of the pulmonary tissue in any of the 30 mice.

Hemosiderin deposits occurred, predominantly in the interstitium and among smooth muscle cells of the bronchial walls, which could be distinguished from immunostaining by the pattern of distribution and by comparison with the corresponding H&E stains. Confirmation was achieved by Prussian blue staining.

It is of note, however, that three mice (10%) developed moderately differentiated invasive adenocarcinoma of the lung. Two mice were in the control group (12 and 15 months), and one was treated with lanreotide (15 months). The tumor cell formations showed invasive growth with destruction of alveolar septa and infiltration in alveolar spaces, as seen in Van Gieson’s stain. They exhibited a papillary and acinar growth pattern, a typical morphology of pulmonary adenocarcinoma. The sizes of the primary tumors were 4 mm, 1 mm, and 1 mm, respectively. There were no lymphatic metastases.

### 3.3. Immunostaining

Neither synaptophysin nor chromogranin positive neuroendocrine cells could be identified in the bronchial wall of any of the examined 30 lung tissues. Therefore, no neuroendocrine cell hyperplasia nor neuroendocrine neoplasia could be diagnosed in these mice.

**Figure 2:**
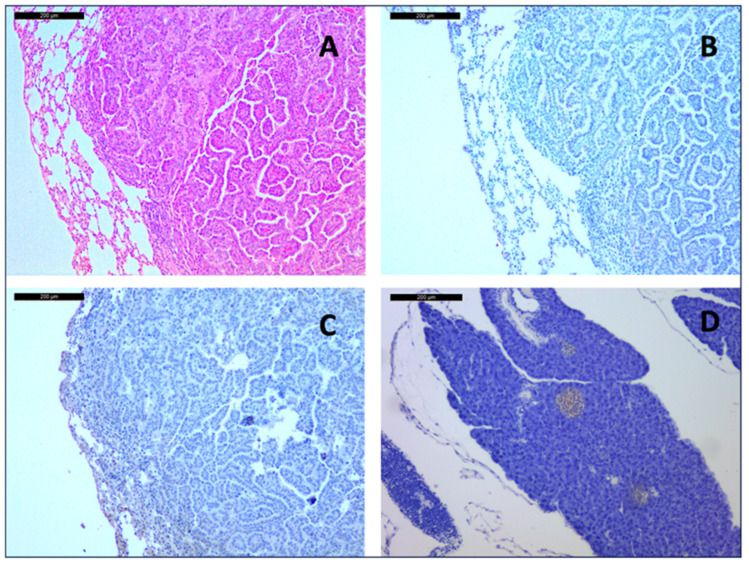
Adenocarcinoma of a 15-month-old placebo-treated mouse at 10× magnification. Hematoxylin and Eosin staining (**A**) shows typical acinar and papillary morphology. Immunohistochemistry showed no expression of Chromogranin A (**B**) or Synaptophysin (**C**), which was strongly expressed in the islets of Langerhans in a pancreatic on-slide control of the same mouse (**D**).

The above-mentioned tumors do not express CgA or synaptophysin, therefore excluding the presence of a neuroendocrine differentiated tumor by WHO definition (Figure 2).

## 4. Discussion

In this study, none of the 15 untreated and 15 SSA-treated heterozygous *Men1* deficient mice showed neuroendocrine cell hyperplasia in the thoroughly examined lung tissue, nor did they develop bronchopulmonary NEN. This is in contrast to our previously reported findings in humans that bronchopulmonary NEN in MEN1 patients might be more common than previously recognized and that multifocal tumorlets and multifocal neuroendocrine cell hyperplasia might represent their precursor lesions [2]. Thus, the goal of preventing the occurrence or growth of neuroendocrine precursor lesions in the animal model by the application of prophylactic SSA could not be achieved.

A weakness of this study was the relatively small number of mice considering the reported penetrance of bronchopulmonary NEN in patients with MEN1 ranging from 2 to 7% [2,3,4]. However, precursor lesions such as neuroendocrine cell hyperplasia and tumorlets may be assumed to be more frequent. Additionally, it was not possible to further investigate the molecular properties of the lung adenocarcinomas due to the small tumor volume and the primary aim of the histopathological workup of neuroendocrine characteristics.

Several earlier studies described the manifestation of neuroendocrine tumors in this animal model [8,12,13,14,15]. The authors concluded that the mouse model mimics the MEN1 syndrome in humans with comparable sites of neuroendocrine tumors, especially in the pancreas and the pituitary, and hyperparathyroidism. However, only one study of a comparable *Men1*-knockout mouse model mentioned findings in the lung [15]. The authors of this study did not find any neuroendocrine tumor, but did find pulmonary adenocarcinoma in 22% of mice. This finding was confirmed in the current study, although the frequency of adenocarcinoma was slightly lower (10%). Like testicular and ovary tumors [13], which are not a known feature of the human MEN1 syndrome, pulmonary adenocarcinoma might be a result of the loss of function specific to the murine tumor suppressor protein menin. Pulmonary adenocarcinoma has not been described to be a part of the human MEN1 phenotype. In the Marburg cohort of 115 patients with MEN1, none had adenocarcinoma of the lung [17]. This finding somehow confirms the results known from some other murine knockout models, such as BRCA1, BRCA2, APC, NF1, and VHL, that all show certain differences compared to the human phenotype [18,19,20]. Therefore, comparisons of animal models with human patients, as well as the effects of certain therapies, generally need to be considered with caution.

The role of SSA in the prophylactic treatment of patients with MEN1 is subject to current clinical investigation. First studies of murine models [8] and a patient series from Italy [9] indicate that SSA has caused a positive effect on the number and growth of small pancreatic NEN. The effect in small pulmonary NENs (<20 mm) has not been investigated to date. Therefore, in this study, we unsuccessfully aimed to determine the prophylactic effect in a preclinical model. It will be the task of a prospective, board-approved observational study to determine whether SSA are justified to slow down the growth of small bronchopulmonary NEN and tumorlets. Furthermore, it has to be clarified whether the application of SSA also prevents the development of the rare aggressive growing NEN in MEN1.

In spite of the expression of SSTR (predominantly SSTR5) in a majority of lung adenocarcinomas, there is no evidence for the preventive or therapeutic efficacy of SSAs in this tumor entity. Small-scale clinical studies on its use for adenocarcinoma of other origin [21,22] and preclinical studies on the use of lanreotide in pancreatic ductal adenocarcinoma [23] showed no effect on tumor size and growth even after in vitro *SSTR2* gene transfer, although *SSTR2* gene transfer itself led to inhibition of tumor growth. Since adenocarcinoma was not considered in the study design, the small number of cases in the current study does not allow any conclusion regarding the effect of lanreotide.

In conclusion, heterozygous *Men1* mutant mice do not develop bronchopulmonary NEN nor precursor lesions, but pulmonary adenocarcinoma in 10% of the individuals.

## Data Availability

Data are contained within the article.

## Abbreviations

The following abbreviations are used in this manuscript:

MEN1	multiple endocrine neoplasia type 1
NEN	neuroendocrine neoplasia
H&E	hematoxillin and eosin
CgA	chromogranin A
ARRIVE	Animal Research: Reporting of In Vivo Experiments
SSA	somatostatin analogues
SSTR	somatostatin receptor
PBS	phosphate buffered saline
HRS	horseradish peroxidase
